# Pathologic Complete Response After Preoperative Ipilimumab and Nivolumab in an HLRCC Patient with Stage III Renal Cell Carcinoma

**DOI:** 10.15586/jkc.v13i2.455

**Published:** 2026-06-01

**Authors:** Neil Rakheja, Payal Kapur, John Zimmerman, Asim Afaq, James Brugarolas, Jeffrey Cadeddu, Hans Hammers

**Affiliations:** 1 St. Mark’s School of Texas, Dallas, TX, USA;; 2 Kidney Cancer Program at Simmons Comprehensive Cancer Center, University of Texas Southwestern Medical Center, Dallas, TX;; 3 Department of Pathology, University of Texas Southwestern Medical Center, Dallas, TX, USA;; 4 Department of Urology, University of Texas Southwestern Medical Center, Dallas, TX;; 5 Cancer Genetics Program of Internal Medicine, University of Texas Southwestern Medical Center, Dallas, TX;; 6 Department of Radiology, University of Texas Southwestern Medical Center, Dallas, TX;; 7 Hematology-Oncology Division of Internal Medicine, University of Texas Southwestern Medical Center, Dallas, TX

**Keywords:** HLRCC, fumarate hydratase-deficient RCC, Immunotherapy, Germline, Kidney

## Abstract

Hereditary leiomyomatosis and renal cell carcinoma (HLRCC) is a rare, aggressive hereditary cancer syndrome caused by germline mutations in the *fumarate hydratase (FH)* gene. Affected patients typically present with renal cell carcinoma (RCC) at a young age and often experience rapidly progressive disease and poor outcomes. Mean survival is significantly shorter for stages III and IV than for stages I and II (15.8 vs 80.7 months), underscoring the need for more effective therapeutic strategies. Here, we report an HLRCC patient with stage III RCC who achieved a pathologic complete response following one cycle of dual immune checkpoint blockade with nivolumab and ipilimumab and remains disease-free 15 months later. This case extends findings from previous reports and suggests that dual checkpoint blockade may result in clinically meaningful activity in a subset of patients.

## Introduction

Hereditary leiomyomatosis and renal cell carcinoma (HLRCC) (OMIM #150800) is a rare syndrome with an autosomal dominant pattern of inheritance, characterized by cutaneous and uterine leiomyomas and an aggressive form of renal cell carcinoma (RCC) with papillary or collecting duct–like morphology (1–4). It is caused by germline mutations in the *fumarate hydratase (FH)* gene, a two-hit tumor suppressor gene located on chromosome 1q42.3–q43 (2–5). These patients are predisposed to other tumors such as pheochromocytoma and paraganglioma, and more recently, the disease was renamed FH Tumor Predisposition Syndrome (6).

The *FH* gene encodes fumarate hydratase, an enzyme that catalyzes the conversion of fumarate to malate in the tricarboxylic acid (TCA) cycle (5). As a result, FH-deficient tumors have impaired oxidative phosphorylation and depend on aerobic glycolysis for survival and proliferation. Fumarate is also an oncometabolite that causes posttranslational cysteine residue modifications and succination, which can be detected by immunohistochemistry for S-(2-succino)cysteine (2SC) (7, 8). A key target is KEAP1, an enzyme implicated in redox homeostasis, which, when succinated, is inactivated, resulting in NRF2 stabilization and the induction of stress response genes (9).

HLRCC-associated RCC tends to occur at a young age, with a mean age of diagnosis around 40 years (range 11–90 years) (10). The lifetime risk of RCC in affected carriers is estimated at 10–20% (11–15). Even small primary tumors (<2 cm) have a propensity for early nodal and distant metastasis (16). The prognosis is poor, with fewer than 30% of patients surviving 5 years after diagnosis (17). Surveillance of at-risk family members is therefore critical for early detection (8, 16).

Histologically, tumors show a papillary to tubulocystic architecture. Tumor cells exhibit an eosinophilic cytoplasm with large viral inclusion–like nucleoli with perinucleolar halos. However, the morphologic spectrum is broad, requiring immunohistochemistry for FH and 2SC for definitive diagnosis (18, 19). In the 2016 WHO classification of renal tumors, HLRCC-associated RCC was recognized as a distinct entity, and in the 2022 update, it was incorporated under the broader category of *FH-*deficient RCC, which includes sporadic cases with biallelic somatic *FH* mutations without family history (16).

Therapeutic advances for HLRCC have been limited. While *FH* inactivation interrupts the TCA cycle, resulting in increased reliance on glycolysis for cell survival and proliferation, glycolysis inhibition failed to show significant activity in an earlier report (20). Conventional therapies for clear cell RCC (ccRCC), the most common RCC subtype, have also shown limited benefit. Tyrosine kinase inhibitors (TKIs) such as pazopanib and cabozantinib have shown modest activity in case reports, but no FDA-approved therapy exists specifically for *FH*-deficient RCC. A recent multicenter Phase II trial showed that bevacizumab plus erlotinib achieves an objective response rate of 72%, with a response duration of 19.3 months (95% CI, 12.9–35.9), but complete responses were observed in only two patients (~5%) (21, 22). Immune checkpoint inhibitors (ICIs), in particular, the combination of nivolumab plus ipilimumab, have resulted in overall survival rates for metastatic ccRCC of ~50% at 5 years, with up to 25% of patients with long-term survival. However, their role in the context of HLRCC is limited. Although prospective data are lacking, FH-deficient RCC frequently demonstrates PD-L1 expression and an inflamed tumor microenvironment (23–25). Clinically, responses have been reported with nivolumab plus ipilimumab (26) and with pembrolizumab monotherapy (27). Gao et al. reviewed published HLRCC cases treated with immunotherapy and concluded that benefit may occur across different *FH* germline variants (28), while Howells et al. described three patients with metastatic FH-deficient RCC, that responded to nivolumab plus ipilimumab (29). Together, these observations suggest that a subset of FH-deficient RCC may be sensitive to immune checkpoint blockade.

Herein, we describe a patient with HLRCC-associated RCC who developed regional nodal metastases while undergoing surveillance and received perioperative therapy with nivolumab plus ipilimumab, with no evidence of disease 15 months later. This case adds to the limited literature, raising the possibility that a subset of patients may derive substantial benefit from checkpoint blockade.

## Case Report

**Figure 1:**
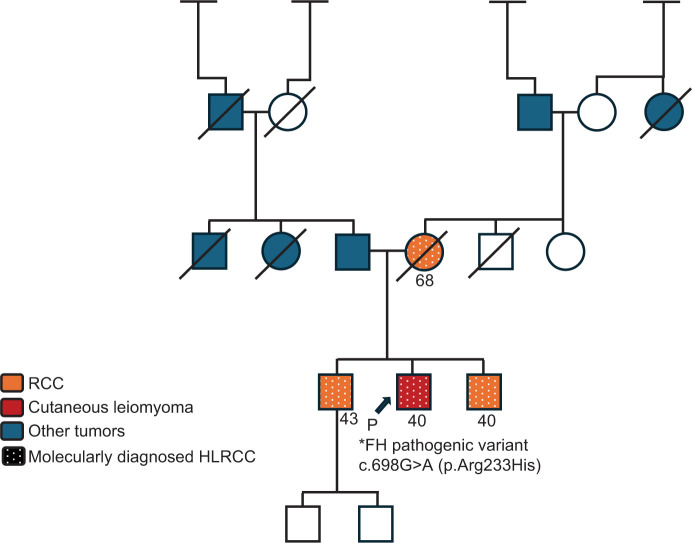
Family pedigree and genomic findings. Pedigree chart showing the proband (arrow) with a pathogenic *FH* germline mutation and affected family members, including his mother and two brothers with HLRCC-associated RCC.

A 40-year-old White male with a family history of HLRCC presented for genetic counseling and surveillance to the UTSW Kidney Cancer Program. His mother had been diagnosed with papillary RCC at the age of 67 and died within a year. His twin brother (diagnosed at age 39) and older brother (diagnosed at age 43) were also recently diagnosed with papillary RCC with molecularly confirmed diagnosis of HLRCC (Figure 1). On physical examination, he had multiple cutaneous leiomyomas. Germline testing confirmed a pathogenic *FH* mutation (c.698G>A [p.Arg233His]). In addition, a variant of uncertain significance in *DICER1* (c.3416-3418delGAA [p.Arg1139del]) was also found.

**Figure 2:**
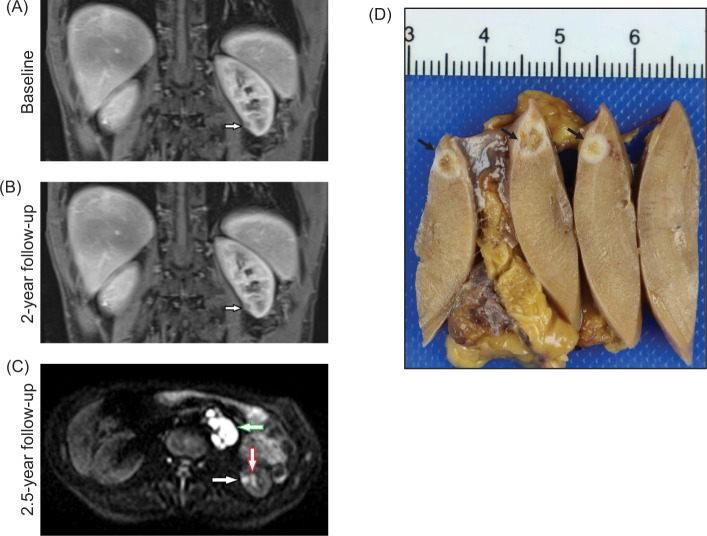
Radiological and macroscopic findings. (A) Coronal post-contrast T1 weighted MR image showing a 9 mm hypoenhancing lesion at the lower pole of the left kidney (white arrow). (B) Coronal post-contrast T1-weighted MR image at 2-year follow-up showing no significant change in size of the hypoenhancing lesion at the lower pole of the left kidney (white arrow). (C) Axial diffusion-weighted MR image at 2.5-years showing restricted diffusion in the same left lower pole lesion (white arrow) and new restricted diffusion (arrow outlined in red) as well as new retroperitoneal lymphadenopathy with restricted diffusion (arrow outlined in green). (D) Macroscopic examination of the left nephrectomy specimen revealed a well-circumscribed tan lesion with a thick fibrotic capsule, in the lower pole measuring 0.6 × 0.4 × 0.4 cm.

Baseline abdominal MRI showed bilateral, small cortical renal cysts without solid masses. He was followed with annual MRIs for 3 years until a 9 mm hypoenhancing lesion was detected in the left lower pole (Figure 2A). Initially managed with surveillance at 6-month intervals, the lesion remained stable for 2 years (Figure 2B). However, 6 months later, an MRI revealed a new area of restricted diffusion next to the nodule in the adjacent parenchyma and a left periaortic lymph node conglomerate (Figure 2C). Core biopsy of a lymph node confirmed the diagnosis of metastatic HLRCC-associated RCC.

Given the aggressive biology of HLRCC-associated RCC and the newly detected regional nodal metastasis, the patient was given one cycle of nivolumab and ipilimumab prior to nephrectomy. The patient underwent a left radical nephrectomy with regional lymph node dissection. Gross examination revealed a small, well-circumscribed tan lesion in the lower pole measuring 0.6 × 0.4 × 0.4 cm (Figure 2D). In addition, a 4.0 × 2.5 × 1.7 cm conglomerate of hilar lymph nodes was provided along with additional interaortocaval and periaortic lymph nodes.

**Figure 3:**
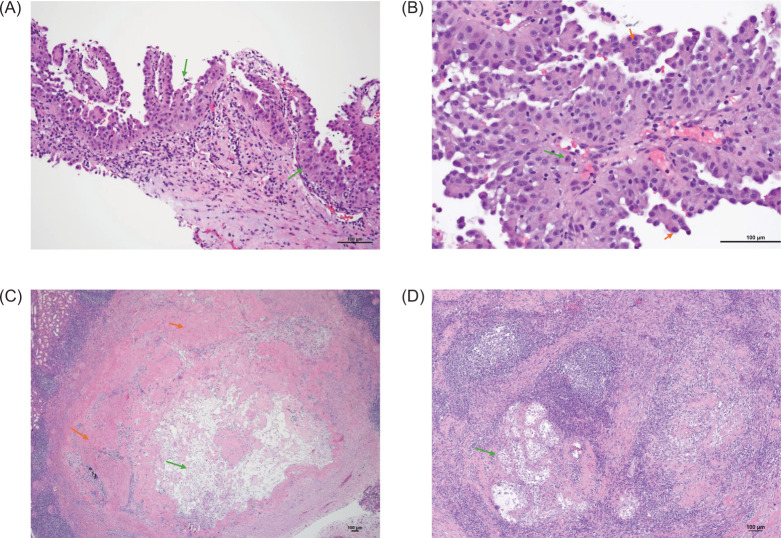
Microscopic features of HLRCC-associated RCC. (A) Core biopsy specimen showing a papillary renal tumor with multilayered neoplastic cells, abundant eosinophilic cytoplasm (green arrow), and prominent eosinophilic nucleoli with perinucleolar clearing (Hematoxylin and eosin (H&E), x100). (B) High-power magnification highlighting the characteristic viral inclusion–like nucleoli (green arrow) with perinucleolar halos (orange arrow (H&E, x200). (C) Nephrectomy specimen demonstrating fibrosis (orange arrow) and chronic inflammation, including histiocytic reaction (green arrow), in the tumor bed without residual viable carcinoma, consistent with a pathologic complete response (H&E, x20). (D) Interaortocaval lymph node showing complete response with replacement by fibrosis and histiocytic reaction (green arrow) (H&E, x40).

Microscopically, the entirely submitted tumor bed showed only fibrous scar tissue and chronic inflammation without viable carcinoma, consistent with a pathologic complete response (CR) (Figure 3). Seven lymph nodes (three hilar and four interaortocaval) also showed similar treatment-related changes with fibrosis, biopsy-site effect, and granulomatous inflammation, but no apparent residual tumor (Figure 3).

**Figure 4:**
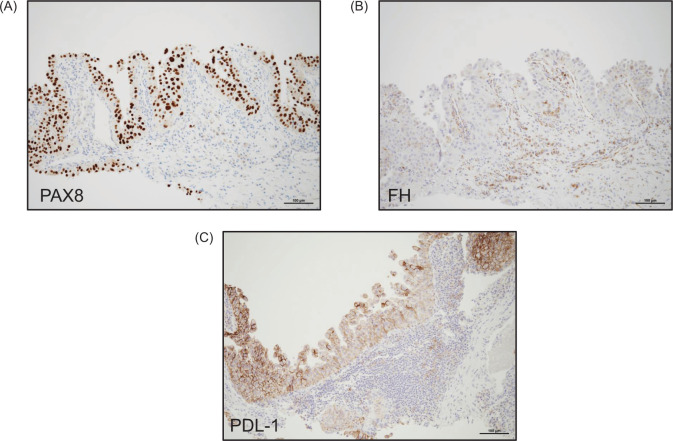
Immunohistochemical profile of HLRCC-associated RCC in core biopsy. (A) Tumor cells showing strong nuclear expression of PAX8, confirming renal epithelial origin (x100). (B) Loss of FH protein expression in tumor cells, with intact staining in surrounding stromal and inflammatory cells (internal positive control; x100). (C) Tumor cells with strong membranous PD-L1 expression in >70% of cells (22C3 antibody; x100).

The pretreatment biopsy from the left periaortic lymph node was concurrently reviewed and showed a PAX8-positive papillary RCC with morphologic features compatible with HLRCC, including multilayered neoplastic cells, eosinophilic cytoplasm, and characteristic viral inclusion-like nucleoli with perinucleolar halos (Figures 3A and 3B). Immunohistochemistry showed loss of FH in tumor cells with preserved staining in stromal and inflammatory elements (Figure 4). Given the remarkable response, PD-L1 IHC was performed (22C3 clone, Dako). This revealed strong membranous PD-L1 reactivity in >70% of tumor cells and ~5% of immune cells (Figure 4C).

Given the pathological complete response and the generally poor outcomes of FH-deficient RCC with lymph node involvement, dual immunotherapy was resumed postoperatively. Following his surgery, he received an additional three cycles of nivolumab and ipilimumab and subsequently 10 cycles of maintenance nivolumab. At 15 months since surgical resection, he remains without radiographic evidence of recurrence.

## Discussion

HLRCC-associated RCC is one of the most aggressive forms of hereditary kidney cancer, with a median overall survival of 16–21 months (17, 25, 30, 31). As our case illustrates, even small primary tumors carry a high metastatic potential, underscoring the importance of vigilant surveillance and the urgent need for timely intervention.

To date, therapeutic options for unresectable FH-deficient RCC remain limited. Retrospective analyses and case reports have described responses to VEGFR TKIs such as pazopanib and cabozantinib, although the durability of response is modest (30, 32). More recently, a prospective Phase 2 study showed that bevacizumab plus erlotinib was associated with an objective response rate of 72%. However, complete responses were observed in only 5% of patients (2 out of 41 patients enrolled) (21). Based on these data, NCCN guidelines recommend bevacizumab plus erlotinib as the preferred regimen for patients with HLRCC-associated RCC (3,3). However, responses remain short-lived for many patients, highlighting the need for alternative strategies.

Immunotherapy, particularly the combination of nivolumab and ipilimumab, represents a therapeutic avenue with the potential for long-term benefit in a subset of patients with ccRCC. The rationale for using these drugs in HLRCC-associated RCC is supported by the frequent expression of PD-L1 in FH-deficient RCC. In a cohort of 13 cases, Alaghehbandan et al. reported PD-L1 positivity in 69% of tumor cells (23), while Yu et al. found high PD-L1 expression in 63% of tumors (n = 19) (24). Somewhat surprisingly PD-L1 expression may be associated with better responses to TKI therapy (34). The combination of nivolumab and ipilimumab has demonstrated durable survival benefit in advanced ccRCC, as shown in the Phase III CheckMate 214 trial (35, 36), but its role in HLRCC remains largely unexplored (26–29).

A review of the literature identified a handful of Stage IV cases of HLRCC-associated RCC achieving durable complete responses with ICIs. Iribe et al. described a patient with metastatic HLRCC who achieved a CR with nivolumab plus ipilimumab (26), and Wang et al. reported a CR with pembrolizumab monotherapy (27). Both tumors exhibited high PD-L1 expression by IHC.

Our case adds to the limited literature and is unique in the pathologic CR after only one cycle of induction immunotherapy in a patient with stage III disease. The optimal management of patients similar to ours is unclear. In clinical practice, most patients with stage III disease are managed surgically. Arguably, the role of surgery is accentuated for non-ccRCC, where systemic therapy has a less-established role. However, HLRCC patients with stage III RCC often develop metastatic disease. Thus, an argument can also be made to explore ICI while the primary tumor is in place, along with infiltrating immune cells. Because the patient had demonstrated substantial response (as shown by a pathologic CR) and tolerated treatment well, postoperative completion of induction combination immunotherapy followed by nivolumab maintenance was chosen.

This case also provides important translational insights. The pretreatment biopsy showed strong PD-L1 expression in >70% of tumor cells, consistent with reports that FH-deficient RCC frequently overexpress PD-L1 (23, 24, 34). The pathologic CR observed in the renal tumor bed and regional lymph nodes underscores the immunogenicity of these tumors and highlights the potential of checkpoint blockade to induce deep and durable remissions in this lethal disease.

Together with previous reports, our report raises the possibility that a subset of FH-deficient RCC may be responsive to ICI, in particular nivolumab and ipilimumab, and we speculate that these tumors may be enriched among those with PD-L1 expression.

## Conclusion

We report a pathologic CR of a stage III HLRCC-associated RCC with regional nodal involvement after a single preoperative cycle of nivolumab plus ipilimumab. Together with prior case reports (26, 27), this case suggests that immune checkpoint blockade may induce deep and potentially durable responses in a subset of patients with FH-deficient RCC. Whether PD-L1 expression enriches for benefit remains unknown and should be considered hypothesis-generating. However, one limitation is reporting bias and how often immunotherapy fails for these patients is unknown. Systematic multi-institutional evaluation of ICI for perioperative and metastatic FH-deficient RCC cases may help define the role of immunotherapy in this disease. While clinical trials in patient populations with rare subtypes are challenging, together with results in the literature, our data supports a more systematic evaluation of ipilimumab and nivolumab in patients with FH-deficient RCC, starting perhaps in the metastatic setting with concomitant evaluation of PD-L1 expression, which could be used as an enrollment criterion or stratification factor.

## Mandatory Disclosure on Use of Artificial Intelligence

The authors declare that no AI-assisted tools were used in the preparation of this manuscript. All references have been manually verified for accuracy and relevance.

## Author Contributions

All authors contributed equally to this article.

## References

[1] Launonen V, Vierimaa O, Kiuru M, Isola J, Roth S, Pukkala E, et al. Inherited susceptibility to uterine leiomyomas and renal cell cancer. Proc Natl Acad Sci USA. 2001;98(6):3387–92. 10.1073/pnas.051633798 11248088 PMC30663

[2] Stewart L, Glenn GM, Stratton P, Goldstein AM, Merino MJ, Tucker MA, et al. Association of germline mutations in the fumarate hydratase gene and uterine fibroids in women with hereditary leiomyomatosis and renal cell cancer. Arch Dermatol. 2008;144(12): 1584–92. 10.1001/archdermatol.2008.517 19075141 PMC2937541

[3] Smit DL, Mensenkamp AR, Badeloe S, Breuning MH, Simon ME, van Spaendonck KY, et al. Hereditary leiomyomatosis and renal cell cancer in families referred for fumarate hydratase germline mutation analysis. Clin Genet. 2011;79(1):49–59. 10.1111/j.1399-0004.2010.01486.x 20618355

[4] Gardie B, Remenieras A, Kattygnarath D, Bombled J, Lefevre S, Perrier-Trudova V, et al. Novel FH mutations in families with hereditary leiomyomatosis and renal cell cancer (HLRCC) and patients with isolated type 2 papillary renal cell carcinoma. J Med Genet. 2011;48(4):226–34. 10.1136/jmg.2010.085068 21398687

[5] Alam NA, Bevan S, Churchman M, Barclay E, Barker K, Jaeger EE, et al. Localization of a gene (MCUL1) for multiple cutaneous leiomyomata and uterine fibroids to chromosome 1q42.3-q43. Am J Hum Genet. 2001;68(5):1264–9. 10.1086/320124 11283798 PMC1226106

[6] Kamihara J, Schultz KA, Rana HQ. FH tumor predisposition syndrome. In: Adam MP, Feldman J, Mirzaa GM, Pagon RA, Wallace SE, Amemiya A, editors. GeneReviews((R)). Seattle (WA): University of Washington, Seattle; 1993. 20301430

[7] Bardella C, El-Bahrawy M, Frizzell N, Adam J, Ternette N, Hatipoglu E, et al. Aberrant succination of proteins in fumarate hydratase-deficient mice and HLRCC patients is a robust biomarker of mutation status. J Pathol. 2011;225(1):4–11. 10.1002/path.2932 21630274

[8] Buelow B, Cohen J, Nagymanyoki Z, Frizzell N, Joseph NM, McCalmont T, et al. Immunohistochemistry for 2-succinocysteine (2SC) and fumarate hydratase (FH) in cutaneous leiomyomas may aid in identification of patients with HLRCC (hereditary leiomyomatosis and renal cell carcinoma syndrome). Am J Surg Pathol. 2016;40(7):982–8. 10.1097/PAS.0000000000000626 26945337

[9] Kinch L, Grishin NV, Brugarolas J. Succination of Keap1 and activation of Nrf2-dependent antioxidant pathways in FH-deficient papillary renal cell carcinoma type 2. Cancer Cell. 2011;20(4):418–20. 10.1016/j.ccr.2011.10.005 22014567 PMC3226726

[10] Hamza A, Sirohi D, Smith SC, Amin MB. Low-grade oncocytic fumarate hydratase-deficient renal cell carcinoma: An update on biologic potential, morphologic spectrum, and differential diagnosis with other low-grade oncocytic tumors. Adv Anat Pathol. 2021;28(6):396–407. 10.1097/PAP.0000000000000321 34561376

[11] Menko FH, Maher ER, Schmidt LS, Middelton LA, Aittomaki K, Tomlinson I, et al. Hereditary leiomyomatosis and renal cell cancer (HLRCC): Renal cancer risk, surveillance and treatment. Fam Cancer. 2014;13(4):637–44. 10.1007/s10689-014-9735-2 25012257 PMC4574691

[12] Schmidt LS, Linehan WM. Hereditary leiomyomatosis and renal cell carcinoma. Int J Nephrol Renovasc Dis. 2014;7:253–60. 10.2147/IJNRD.S42097 25018647 PMC4074185

[13] Toro JR, Nickerson ML, Wei MH, Warren MB, Glenn GM, Turner ML, et al. Mutations in the fumarate hydratase gene cause hereditary leiomyomatosis and renal cell cancer in families in North America. Am J Hum Genet. 2003;73(1):95–106. 10.1086/376435 12772087 PMC1180594

[14] Forde C, Lim DHK, Alwan Y, Burghel G, Butland L, Cleaver R, et al. Hereditary leiomyomatosis and renal cell cancer: Clinical, molecular, and screening features in a cohort of 185 affected individuals. Eur Urol Oncol. 2020;3(6):764–72. 10.1016/j.euo.2019.11.002 31831373

[15] Scharnitz T, Nakamura M, Koeppe E, Henry ML, Lowe L, Else T, et al. The spectrum of clinical and genetic findings in hereditary leiomyomatosis and renal cell cancer (HLRCC) with relevance to patient outcomes: A retrospective study from a large academic tertiary referral center. Am J Cancer Res. 2023;13(1):236–44. 36777509 PMC9906083

[16] Moch H, Amin MB, Berney DM, Comperat EM, Gill AJ, Hartmann A, et al. The 2022 World Health Organization classification of tumours of the urinary system and male genital organs—Part A: Renal, penile, and testicular tumours. Eur Urol. 2022;82(5):458–68. 10.1016/j.eururo.2022.06.016 35853783

[17] Patel VM, Handler MZ, Schwartz RA, Lambert WC. Hereditary leiomyomatosis and renal cell cancer syndrome: An update and review. J Am Acad Dermatol. 2017;77(1):149–58. 10.1016/j.jaad.2017.01.023 28314682

[18] Merino MJ, Torres-Cabala C, Pinto P, Linehan WM. The morphologic spectrum of kidney tumors in hereditary leiomyomatosis and renal cell carcinoma (HLRCC) syndrome. Am J Surg Pathol. 2007;31(10):1578–85. 10.1097/PAS.0b013e31804375b8 17895761

[19] Kapur P, Amin MB, Chen Y-B, Cheville J, Hes O, Rakheja D, et al. Recent updates in pathology of renal cell carcinoma: Société Internationale d’Urologie; 2022. Available from: https://www.siu-urology.org/themes/web/assets/files/2023/icud_webpage/2nd-wuof-siu-icud-kidney-cancer-final-october17-2022.pdf

[20] Yamasaki T, Tran TA, Oz OK, Raj GV, Schwarz RE, Deberardinis RJ, et al. Exploring a glycolytic inhibitor for the treatment of an FH-deficient type-2 papillary RCC. Nat Rev Urol. 2011;8(3):165–71. 10.1038/nrurol.2010.234 21304509 PMC3055922

[21] Srinivasan R, Gurram S, Singer EA, Sidana A, Al Harthy M, Ball MW, et al. Bevacizumab and erlotinib in hereditary and sporadic papillary kidney cancer. N Engl J Med. 2025;392(23):2346–56. 10.1056/NEJMoa2200900 40532152

[22] Choi Y, Keam B, Kim M, Yoon S, Kim D, Choi JG, et al. Bevacizumab plus erlotinib combination therapy for advanced hereditary leiomyomatosis and renal cell carcinoma-associated renal cell carcinoma: A multicenter retrospective analysis in Korean patients. Cancer Res Treat. 2019;51(4):1549–56. 10.4143/crt.2019.086 30913859 PMC6790829

[23] Alaghehbandan R, Stehlik J, Trpkov K, Magi-Galluzzi C, Condom Mundo E, Pane Foix M, et al. Programmed death-1 (PD-1) receptor/PD-1 ligand (PD-L1) expression in fumarate hydratase-deficient renal cell carcinoma. Ann Diagn Pathol. 2017;29:17–22. 10.1016/j.anndiagpath.2017.04.007 28807336

[24] Yu Y, Shen Q, Xia M, Huang C, Li X, He S, et al. Fumarate hydratase-deficient renal cell carcinoma: High intratumoral and peritumoral CD4-positive T cell infiltration density and high PD-L1 expression. World J Urol. 2025;43(1):567. 10.1007/s00345-025-05941-6 40982012

[25] Sun G, Zhang X, Liang J, Pan X, Zhu S, Liu Z, et al. Integrated molecular characterization of fumarate hydratase-deficient renal cell carcinoma. Clin Cancer Res. 2021;27(6):1734–43. 10.1158/1078-0432.CCR-20-3788 33414138

[26] Iribe Y, Furuya M, Shibata Y, Yasui M, Funahashi M, Ota J, et al. Complete response of hereditary leiomyomatosis and renal cell cancer (HLRCC)-associated renal cell carcinoma to nivolumab and ipilimumab combination immunotherapy by: A case report. Fam Cancer. 2021;20(1):75–80. 10.1007/s10689-020-00195-0 32666341

[27] Wang T, Huang Y, Huang X, Lv Z, Tian S, Ma X, et al. Complete response of hereditary leiomyomatosis and renal cell cancer (HLRCC)-associated renal cell carcinoma to pembrolizumab immunotherapy: A case report. Front Oncol. 2021;11:735077. 10.3389/fonc.2021.735077 34722283 PMC8554149

[28] Gao F, Gu D, Zhang H, Shi C, Du F, Zheng B, et al. Case report: Response to immunotherapy and association with the fh gene in hereditary leiomyomatosis and renal cell cancer-associated renal cell cancer. BMC Med Genomics. 2024;17(1):215. 10.1186/s12920-024-01957-w 39160519 PMC11331603

[29] Howells E, Wigston L, Mackie G, Tran B, Nott L. Advanced fumarate hydratase-deficient renal cell carcinoma responding to combination immune checkpoint inhibitors. Can J Urol. 2023;30(3):11558–61. 37344468

[30] Muller M, Ferlicot S, Guillaud-Bataille M, Le Teuff G, Genestie C, Deveaux S, et al. Reassessing the clinical spectrum associated with hereditary leiomyomatosis and renal cell carcinoma syndrome in French FH mutation carriers. Clin Genet. 2017;92(6):606–15. 10.1111/cge.13014 28300276

[31] Sanchez-Heras AB, Castillejo A, Garcia-Diaz JD, Robledo M, Teule A, Sanchez R, et al. Hereditary leiomyomatosis and renal cell cancer syndrome in Spain: Clinical and genetic characterization. Cancers (Basel). 2020;12(11):3277. 10.3390/cancers12113277 33167498 PMC7694543

[32] Park I, Shim YS, Go H, Hong BS, Lee JL. Long-term response of metastatic hereditary leiomyomatosis and renal cell carcinoma syndrome associated renal cell carcinoma to bevacizumab plus erlotinib after temsirolimus and axitinib treatment failures. BMC Urol. 2019;19(1):51. 10.1186/s12894-019-0484-2 31182090 PMC6558845

[33] Motzer RJ, Jonasch E, Agarwal N, Alva A, Bagshaw H, Baine M, et al. NCCN Guidelines(R) insights: Kidney cancer, Version 2.2024. J Natl Compr Canc Netw. 2024;22(1):4–16. 10.6004/jnccn.2024.0008 38394781

[34] Bai J, Huang J, Ye Y, Lin J, Wen Y, Cai Z, et al. Clinicopathologic and prognosis of 30 patients with FH-deficient renal cell carcinoma: A single-center retrospective study. BMC Cancer. 2025;25(1):1152. 10.1186/s12885-025-14562-6 40624460 PMC12235827

[35] Motzer RJ, Tannir NM, McDermott DF, Aren Frontera O, Melichar B, Choueiri TK, et al. Nivolumab plus ipilimumab versus sunitinib in advanced renal-cell carcinoma. N Engl J Med. 2018;378(14):1277–90. 10.1056/NEJMoa1712126 29562145 PMC5972549

[36] Tannir NM, Albiges L, McDermott DF, Burotto M, Choueiri TK, Hammers HJ, et al. Nivolumab plus ipilimumab versus sunitinib for first-line treatment of advanced renal cell carcinoma: Extended 8-year follow-up results of efficacy and safety from the phase III CheckMate 214 trial. Ann Oncol. 2024;35(11):1026–38. 10.1016/j.annonc.2024.07.727 39098455 PMC11907766

